# Supplementary material for: Clinical and Cytokine Profile in Patients with Early and Late Onset Meniere Disease
Source: J Clin Med. 2021 Sep 7;10(18):4052. doi: 10.3390/jcm10184052 (PMC8472422; doi:10.3390/jcm10184052)
Supplement: Supplementary file 1 [file jcm-10-04052-s001.zip › jcm-1350628-supplementary.pdf]

**Table S1.** Clinical features and basal levels of IL-1β cytokine in migraine patients (n = 64).

| Patient | Gender | Age | Age of onset | Duration of Disease | Type of migraine | Hearing Loss | IL-1ß levels |
|---|---|---|---|---|---|---|---|
| 1 | woman | 25 | 20 | 5 | with aura | No | 2.65 |
| 2 | woman | 26 | 12 | 14 | without aura | No | 1.24 |
| 3 | man | 21 | 15 | 6 | with aura | No | 4.21 |
| 4 | man | 17 | 12 | 5 | without aura | No | 4.40 |
| 5 | man | 53 | 12 | 40 | with aura | No | **4.78** |
| 6 | man | 55 | 15 | 40 | without aura | No | 0 |
| 7 | man | 33 | 12 | 11 | without aura | No | **5.02** |
| 8 | man | 39 | 32 | 7 | with aura | No | **5.77** |
| 9 | woman | 24 | 10 | 14 | without aura | No | 0.43 |
| 10 | man | 20 | 13 | 7 | with aura | No | 0 |
| 11 | man | 30 | 12 | 18 | with aura | No | 0 |
| 12 | man | 39 | 13 | 26 | without aura | No | 0 |
| 13 | woman | 47 | 20 | 27 | without aura | No | 0.02 |
| 14 | woman | 15 | 13 | 2 | with aura | No | 0 |
| 15 | woman | 34 | 33 | 1 | without aura | No | **26.31** |
| 16 | woman | 38 | 33 | 5 | with aura | No | **6.88** |
| 17 | woman | 40 | 29 | 11 | with aura | Yes | **474.76** |
| 18 | woman | 32 | 15 | 17 | without aura | No | **7.40** |
| 19 | woman | 34 | 20 | 13 | with aura | No | 1.34 |
| 20 | woman | 25 | 22 | 3 | with aura | No | 2.53 |
| 21 | woman | 20 | 14 | 6 | without aura | No | **6.03** |
| 22 | woman | 41 | 38 | 3 | without aura | No | 3.49 |
| 23 | woman | 59 | 14 | 45 | with aura | No | **29.77** |
| 24 | woman | 27 | 25 | 2 | with aura | No | **69.34** |
| 25 | woman | 37 | 31 | 6 | without aura | No | **1149.73** |
| 26 | woman | 34 | 31 | 3 | with aura | Yes | **155.70** |
| 27 | woman | 25 | 18 | 7 | without aura | No | 1.13 |
| 28 | man | 25 | 8 | 17 | with aura | No | 1.74 |
| 29 | man | 35 | 15 | 20 | without aura | No | 1.54 |
| 30 | woman | 45 | 20 | 25 | without aura | No | 1.74 |
| 31 | man | 43 | 35 | 8 | with aura | No | 2.92 |
| 32 | woman | 51 | 47 | 4 | without aura | No | **8.58** |
| 33 | woman | 24 | 8 | 16 | without aura | No | 2.14 |
| 34 | woman | 14 | 14 | 1 | with aura | No | 1.34 |
| 35 | man | 16 | 7 | 9 | without aura | No | 2.14 |
| 36 | man | 28 | 25 | 3 | without aura | No | 1.54 |
| 37 | woman | 38 | 22 | 16 | with aura | No | 1.34 |
| 38 | woman | 42 | 20 | 22 | without aura | No | 1.74 |

| | | | | | | | |
|---|---|---|---|---|---|---|---|
| 39 | woman | 61 | 30 | 31 | without aura | No | 2.53 |
| 40 | woman | 38 | 18 | 20 | with aura | No | 1.13 |
| 41 | woman | 37 | 16 | 21 | without aura | No | 0.72 |
| 42 | woman | 57 | 30 | 27 | with aura | No | **6.30** |
| 43 | woman | 43 | 38 | 5 | without aura | No | 0.93 |
| 44 | man | 68 | 31 | 37 | without aura | No | 0.72 |
| 45 | woman | 43 | 27 | 16 | without aura | No | 1.54 |
| 46 | woman | 63 | 45 | 18 | without aura | No | **15.71** |
| 47 | woman | 31 | 27 | 4 | without aura | No | 0.72 |
| 48 | man | 57 | 40 | 17 | without aura | No | 0.93 |
| 49 | woman | 20 | 18 | 2 | without aura | No | 0 |
| 50 | woman | 31 | 27 | 4 | with aura | No | **4.82** |
| 51 | woman | 50 | 30 | 20 | without aura | No | 1.34 |
| 52 | woman | 41 | 40 | 1 | with aura | No | **6.85** |
| 53 | woman | 50 | 46 | 4 | with aura | No | 1.74 |
| 54 | woman | 29 | 24 | 5 | without aura | No | 0.72 |
| 55 | woman | 54 | 48 | 6 | without aura | No | 1.34 |
| 56 | woman | 54 | 34 | 20 | without aura | No | 1.24 |
| 57 | woman | 60 | 20 | 40 | with aura | No | 2.73 |
| 58 | woman | 42 | 22 | 20 | without aura | No | 4.26 |
| 59 | man | 32 | 24 | 8 | with aura | No | 3.30 |
| 60 | woman | 67 | 45 | 22 | without aura | No | 2.53 |
| 61 | man | 52 | 37 | 15 | without aura | No | 0.0 |
| 62 | woman | 39 | 34 | 5 | without aura | No | **5.75** |
| 63 | woman | 52 | 32 | 20 | without aura | Yes | 3.69 |
| 64 | woman | 55 | 36 | 19 | without aura | No | 0.93 |

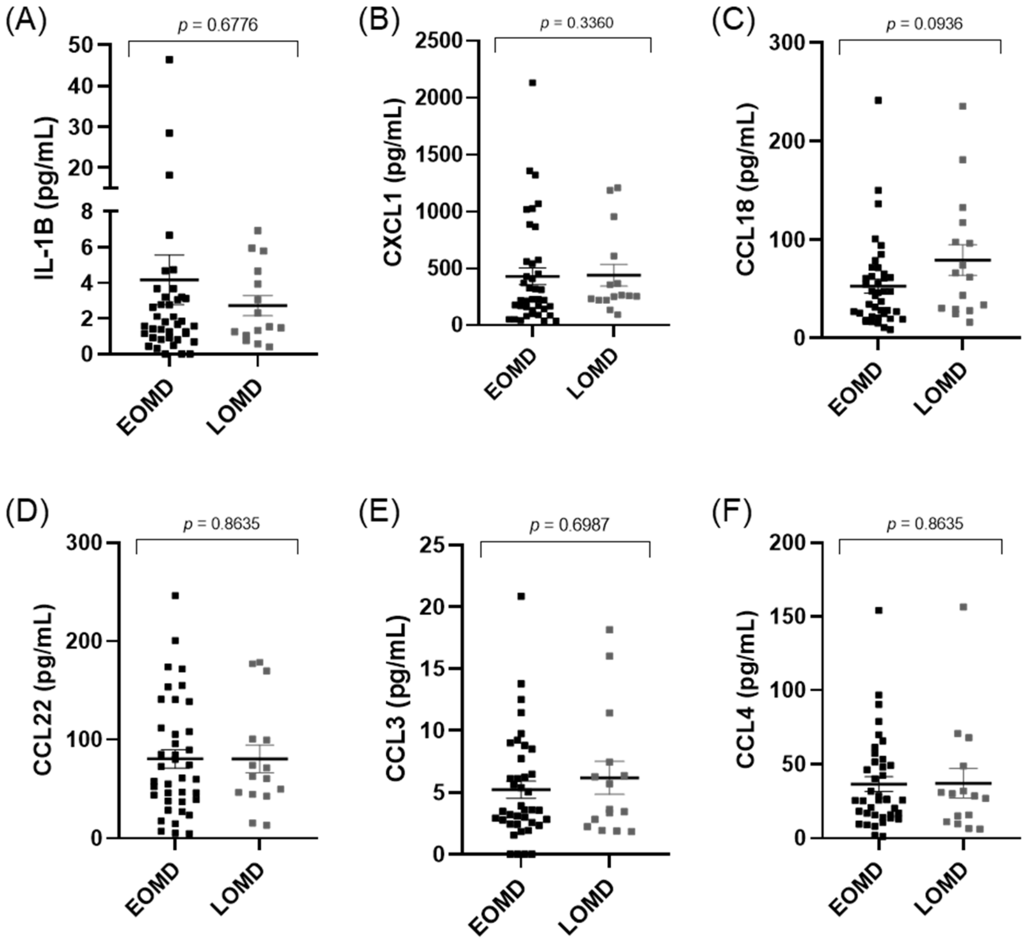


**Figure S1.** Scattered plot showing cytokines levels in the supernatant of PBMCs from patients with EOMD and LOMD. IL-1β (**A**), CXCL1(**B**), CCL18 (**C**), CCL22 (**D**), CCL3 (**E**), CCL4 (**F**).

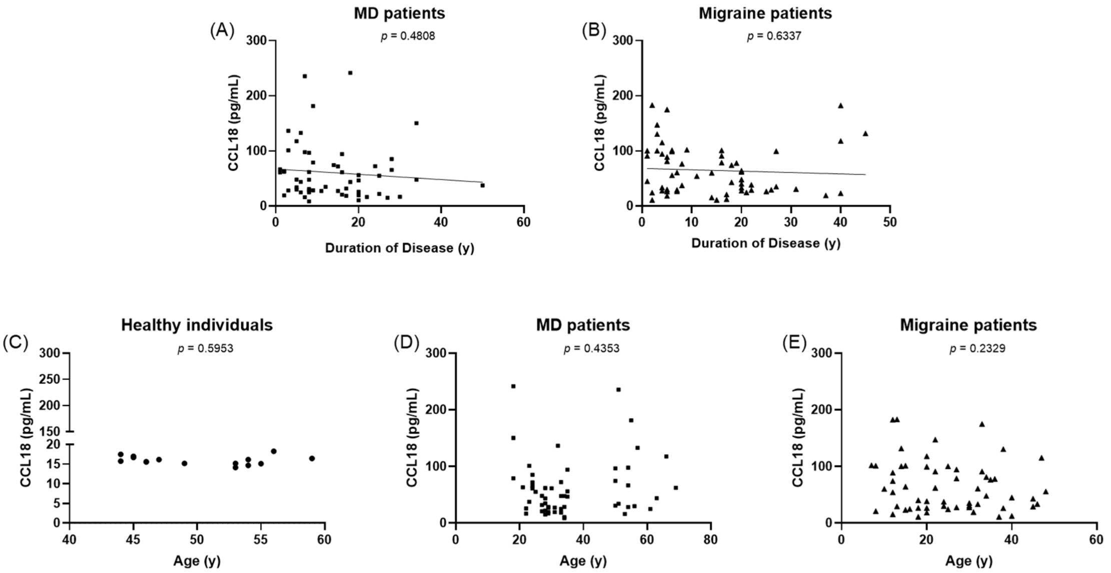


**Figure S2.** Scattered plots showing CCL18 levels as a function of the duration of disease for MD (A) and migraine patients (B) or the age of individuals in healthy controls (C), patients with MD (D) and migraine (E). No association was found between CCL18 levels and duration of disease or age of individuals.
